# A Hierarchy of Power: The Place of Patient and Public Involvement in Healthcare Service Development

**DOI:** 10.3389/fsoc.2019.00038

**Published:** 2019-05-08

**Authors:** Alison O'Shea, Annette L. Boaz, Mary Chambers

**Affiliations:** Centre for Health and Social Care Research, Kingston University & St. George's University, London, United Kingdom

**Keywords:** patient and public involvement, healthcare, power, hierarchy, influence, lay members, public members, professionals

## Abstract

Amidst statutory and non-statutory calls for effective patient and public involvement (PPI), questions continue to be raised about the impact of PPI in healthcare services. Stakeholders, policy makers, researchers, and members of the public ask in what ways and at what level PPI makes a difference. Patient experience is widely seen as an important and valuable resource to the development of healthcare services, yet there remain legitimacy issues concerning different forms of knowledge that members of the public and professionals bring to the table, and related power struggles. This paper draws on data from a qualitative study of PPI in a clinical commissioning group (CCG) in the UK. The study looked at some of the activities in which there was PPI; this involved researchers conducting observations of meetings, and interviews with staff and lay members who engaged in CCG PPI activities. This paper explores power imbalances when it comes to influencing the work of the CCG mainly between professionals and members of public, but also between different CCG staff members and between different groups of members of public. The authors conclude that a hierarchy of power exists, with some professionals and public and lay members afforded more scope for influencing healthcare service development than others—an approach which is reflected in the ways and extent to which different forms and holders of knowledge are viewed, managed, and utilized.

## Introduction

It is widely accepted that quality in the delivery of healthcare is more than purely good clinical care. Quality is now defined to include dimensions such as clinical effectiveness, safety, and patient-centredness (Institute of Medicine (US) Committee on Quality of Health Care in America, 2001). The experience and voice of the patient has increasingly become integrated as a core dimension of health care consultation and planning. The idea is that patient and public involvement will improve quality and implementation of healthcare services, address population expectations and needs and foster healthcare choices and shared decision-making (Boivin et al., 2010).

Patient and public involvement (PPI) in healthcare services has become an international phenomenon over recent years in western and developing countries (Oliver et al., 2007). A PPI working group created in 2007 by the Guideline International Network Patient and Public Involvement Working Group (G-I-N PUBLIC) aims to support PPI globally in the development and implementation of clinical practice guidelines (Boivin et al., 2010). The UK is viewed as one of the “pioneers” in demonstrating a national commitment to public involvement (Gauvin et al., 2010). Regulations stipulate that all NHS organizations must have PPI in the planning, development, and operational aspects of healthcare services (Barnes and Schattan Coelho, 2009).

Statutory policy produces guidance on patient and public participation for commissioners of health services (NHS England, patient, and public participation policy)^1^. Statutory guidance, however, is open to interpretation (Martin, 2008a) which often results in contrasting approaches to PPI and outcomes. Madden and Speed point out: “At its best, PPI may have the potential for increased democratic accountability, for improving health outcomes, and for addressing the social determinants of health, through for example, improved understanding of different cultures of research and engagements with evidence. At its worst, however, PPI runs the risk of being insignificant, tokenistic, and overly managerialist” (2017, p. 1).

Attempts to broaden and strengthen PPI continue in response to calls for more effective involvement. Frameworks have been developed for exploring the nature of PPI in the context of different approaches used and the differences between professionals and the public in relation to the challenges, demands and expectations around PPI (e.g., Tritter, 2009; Gibson et al., 2012). However, questions continue to be raised about the level and impact of PPI in decision-making and more recently research and literature has highlighted the importance addressing various challenges associated to these issues (e.g., Mockford et al., 2011; Staniszewska et al., 2011; Brett et al., 2012). Moreover, whilst patient experience is viewed as an important and valuable resource to the development of healthcare services, there are concerns about the legitimacy of the type of knowledge that patients and members of the public possess and are therefore able to contribute to healthcare decision-making (Daykin et al., 2007; Martin, 2008b; Boivin et al., 2010).

One of the challenges facing effective PPI relates to the differences between professionals and patients and the public in terms of motivations, expectations, and perceptions of PPI (Rise et al., 2011). Calls have been made for “professionals and users […] to reconceptualize the traditional category of patient to one that understands that service users can contribute to service planning and development” (Petsoulas et al., 2014, p. 10).

This paper stems from a study which set out to explore PPI in a clinical commissioning group (CCG). Clinical commissioning groups are clinically-led statutory NHS bodies. Since April 2013, following a shift in commissioning powers from the former Primary Care Trusts, CCGs have held responsibility for commissioning secondary and community care services for their local populations. Clinical commissioning groups control around two-thirds of the NHS budget. All general practices in England are legally obliged to be a member of a CCG (Naylor et al., 2013; p. ix).

The principles of PPI have been formally incorporated within the structure of CCGs through regulations which stipulate that the governing body of each CCG must include eight statutory roles, of which two must be occupied by lay members. Involvement from public and patient representatives is emphasized within reformed commissioning structures and procedures which require CCGs to liaise with Health and Well-being Boards to plan and deliver services. Healthwatch representatives form part of these Health and Well-being Boards and are described as the “patient voice” or “consumer champion” (Department of Health, 2012).

The CCG PPI study revealed that a key dimension of PPI related to a system of stratification within which individuals occupied positions that reflected their capacity to influence the work of the CCG. Stratification systems are a common feature of developed societies where a dominant hierarchy exists to maintain stability. As such, stratification engenders inequalities around power and other valued resources (Cheng et al., 2013). Raphael and Bryant (2015) provide a useful characterization of stratification: “In addition to affecting the social determinants of health, stratification is related to the power and ability of those so stratified to influence public policy” (Raphael and Bryant, 2015, p. 248).

Stratification constitutes a hierarchy which distinguishes between individuals on the basis of power to influence. Variations in power status within healthcare structures are widely documented, with reference to paid professionals occupying more dominant positions than members of public (e.g., Martin, 2008b; Ocloo and Matthews, 2016).

Foucault (1972) describes power as typically residing in medical structures, institutions, and discourses. The growth of PPI and the resulting increased potential it brings for patients and the public to have a voice in healthcare decision-making might potentially counteract this view. However, the question in reality is whether the growth of PPI translates into patients and the public having power to influence healthcare service development. Themes of power, dominance, and hierarchy are prevalent analytical terms in sociological studies of health care, and Foucault's concepts around power and knowledge can be applied to traditional views of the doctor-patient interactions. In historical contexts, Foucault theorized power of knowledge as embedded in dominant discourses and systems, and viewed critiques of knowledge and truths as both pervasive and dominant. Under such an analysis of power and hierarchy, the medical profession maintained the upper hand by having greater knowledge, expertise, prestige, organizational support, and stability. The patient/public with historically less power such as women, minority groups, and the poor would have been more vulnerable playing “second fiddle” to medical authority and lacked the resources to question medical decisions or challenge prescribed care (Foucault, 2003). However, as the modern day patient/public has become better informed about illnesses and forms of treatment, they have become self-advocates for their own health care and perhaps, in this sense, as a consequence have acquired the potential to narrow the power disparity.

There are various definitions of power that have emanated from different theoretical and conceptual perspectives. A neutral meaning of power cannot be found, since the meaning of power is always embedded in a theoretical context (Guzzini, 2005). It is not the intention here to provide an in-depth discussion of the different perspectives of the meaning of power. However, one example of a classical sociological concept of power comes from Weber's definition, presented by Rutar (2017):

“For him (Weber, 1978 [1922], p. 53) power is, as is well known, “the probability that one actor within a social relationship will be in a position to carry out his own will despite resistance, regardless of the basis on which this probability rests.” This can be, and usually is, further condensed. Social power is simply the ability of agent A to influence agent B in such a way (with the help of either personal or impersonal means) that agent B does something he/she otherwise would not have done, or does not do something he/she otherwise would have done (cf. Dahl, 1961)” (2017, p. 153).

## Methods

The aim of this paper is to explore the differences between individuals in their potential to influence the work of the CCG and to consider these differences in terms of the positions they occupy in a hierarchical structure.

The use of the term “public member” refers to a member of public; the term “lay member” refers to a member of public who is a member of a formal PPI group.

### Study Design

This study forms part of a wider research project exploring PPI in a CCG in England. It is a single case study set in a large, diverse inner city. The study used a qualitative approach drawing on ethnographic methods. Ethnographic research “seeks to understand people's opinions, beliefs, motivations, interactions, and the structures in which they are involved or are influenced by, and above all, the social contexts in which people live and interact […]. [Ethnographic research] observes what people do in their everyday practices, and tries to understand the motivations and explanations for people's actions.” (Potrata, 2005, p. 131).

PPI in the CCG comprised GP surgery patient groups, public consultations, public attendance at CCG board public meetings, and various clinical reference groups (CRGs). Our study explored two of these activities: (i) CCG board public meetings and (ii) the CRG for PPI (PPI CRG). These settings were identified by a PPI lay member of the CCG board who was involved in developing the study.

### Data Collection

Data were collected over an 18 month period between February 2014 and August 2015 and methods comprised observations, informal interactions, interviews, and a focus group.

#### Observations

Researchers made handwritten notes of observations which were entered as soon as possible after each meeting onto a data collection tool (form) that was designed for the study. The tool enabled researchers to document the type of meeting, number of people present and their roles, diagram/notes on physical layout, agenda items discussed, and researchers' general notes.

##### Clinical commissioning group board public meetings

CCG board public meetings were held monthly on a weekday morning in a CCG meeting room. Observations of 14 meetings were conducted. Meetings lasted two and a half hours; this constituted ~35 h of observation. Researchers considered it necessary to carry out this number of observations because meetings covered a range of topics which often varied from month to month. This generated attendance from different public and staff members depending on the agenda items under discussion.

The board was made up of 15 voting members and six non-voting members. Voting members included two lay members, one with responsibility for governance and one with responsibility for PPI. Other voting members comprised clinicians (GPs and a secondary care doctor), a registered nurse and managers of finance/accounts. Non-voting board members included directors of services and a Healthwatch representative.

##### Patient and public involvement clinical reference group meetings

PPI CRG meetings took place bimonthly on a weekday afternoon in a CCG meeting room. Observations of 10 of these meetings were carried out. Meetings lasted ~3 h, amounting to 30 h of observation.

The PPI CRG was relatively newly developed and observing 10 meetings over an 18 month period enabled researchers to gain insight into the nature and progression of the group and the relationships within. There were 11 lay members of the group, made up of individuals and community representatives, voluntary and community sector representatives, locality representatives, and a lay member chair of the group (who was also one of the voting lay members on the CCG board). The group comprised five staff members: a clinical lead, a PPI manager, an engagement manager, an administrator and a CCG board member with a remit for PPI. The CCG board member's attendance reduced regarding the amount of time spent in meetings and ceased altogether less than half way through observations. This was reportedly because their attendance was seen by the CCG as necessary only during the early stages of the group's development to provide support until the group had become more established.

Meeting attendance numbers varied (in terms of both staff and lay member attendance), with a minimum of five members in just one meeting observed and a maximum of 15 in another.

#### Informal Interactions

Informal interactions between researchers and meeting attendees often occurred following CCG board public meetings and PPI CRG meetings. Interactions provided valuable insight into the views, beliefs and experiences of public, lay and staff members in terms of the respective meeting and PPI itself. Handwritten notes of interactions were added to the relevant observation notes on the data collection tool.

#### Interviews

A total of 14 interviews, both face to face and by telephone (according to the preference of interviewees), were carried out with staff, public, and lay members. These comprised three with CCG board members, one with a public member who regularly attended CCG board public meetings, two with PPI CRG staff, and eight with PPI CRG lay members (Table 1).

**Table 1:** Number of interviews conducted with CCG staff and public/lay members.

**CCG staff**		**Public/lay members**	
Board members	*n* = 3	Public member attendee of CCG board meeting	*n* = 1
PPI CRG lead/manager	*n* = 2	PPI CRG lay members!!! (inc lay chair board member)	*n* = 8

Interview schedules for public and lay members addressed the following areas: how and why they became involved in the CCG and associated expectations; the PPI role; representation as a lay/public member; CCG support for and commitment to PPI; impact/influence of PPI. Public members who attended CCG board public meetings were also asked about PPI in meetings and the structure and content of meetings. Staff member interview schedules explored issues around the importance, benefits and challenges, and influence of PPI.

#### Focus Group

A focus group took place 12 months into data collection activities with five PPI CRG lay members. In particular, discussion focused on issues around representation. Three focus group lay members also took part in the interviews.

### Data Analysis

Analysis was an iterative process carried out at different stages starting from the collection of data during observations through to writing up findings of the study. Analysis of observation and interaction notes and interview and focus group transcripts took place using a thematic framework approach (Pope et al., 2000). Data were coded into themes from which interpretations were generated. This process was carried out inductively, identifying key issues, concepts and themes emerging from data, and deductively in line with interview and focus group schedules. During data collection activities and early coding we observed that power imbalance was a key feature within the different data sources: observations of meetings, interviews, informal interactions and the focus group. Ongoing coding and analysis generated the themes presented in Table 2. The three layers represent the final set of themes and codes underpinning our analysis: Overarching theme (power, control, PPI impact); Organizing theme (PPI, power to influence, time, meeting arrangements, recruitment, knowledge, accountability, and feedback); Components of organizing theme.

**Table 2:** Data coding themes.

**Overarching theme**	**Organizing theme**	**Components of organizing theme**
Power	Power over PPI	Type of involvement!!! Level of involvement!!! Timing of involvement!!! Place of PPI!!! PPI CRG role unclear!!! Challenges—limited resources for PPI in commissioning
	Power to influence	Unequal between professionals and public/lay members!!! Unequal between different public/lay members!!! Decision-making!!! Status of relationships
Control	Time (CCG board public meetings)	Short of time—PPI reduced!!! Changes to PPI timeslot!!! Written public questions not always responded to
	Meeting arrangements	Time, venue, frequency!!! Agenda setting and discussion!!! Leadership of meetings—staff; lay member!!! Supporting PPI
	Recruitment	PPI CRG membership and!!! skills/attributes required, determined by the CCG!!! Leadership of PPI CRG
PPI Impact	Knowledge	Capacity to influence/make a difference!!! Legitimacy!!! Skills and experience!!! Value of PPI
	Accountability and feedback	Role of PPI unclear!!! Feedback not shared with PPI CRG!!! Accountability—one way!!! Monitoring/evaluation of PPI

Two researchers independently carried out analysis of half the data. Following discussion and agreement about the coding and subsequent themes identified, one researcher continued the process across the other half of data. Papers and documents from meetings observed were collated and used for reference during data analysis.

### Patient and Public Involvement

The original idea for this study emerged from a discussion between a lay member of the CCG board (who was our gatekeeper to the meetings observed) and the investigators. Many of our research participants were lay and public members. Subsamples of three lay members volunteered and were subsequently involved in the design of the research, data collection, and analysis.

### Ethics

This study was granted ethical approval on 14/02/14 by the East of Scotland Research Ethics Service.

## Results

Findings from our data reveal that the CCG retained power and control over PPI in different ways.

### Clinical Commissioning Group Board Public Meetings

CCG board public meetings were intended to enable public participation. Through open discussion, meetings would inform the public of service developments and provide opportunity for questions about issues under debate. Whilst discussions about service development took place between board members, there appeared to be little opportunity for public participation.

#### Control

The CCG board controlled all the meeting and PPI arrangements and agenda items for discussion. For one public member there was:

a slight tendency to listen to things that fit into their agenda—slightly […] if someone raises something pertinent, they might be useful to the board with their knowledge then maybe they should be following up on that rather than seeing it as “oh god, that's something else to do and that's another problem you've given us.”

At times meetings did not feel open to public participation:

[…] so if [they] think it's not a relevant question, [they'll] close down quite quickly I think on the question. And [they are] very clear of the direction [they] want to go in and I don't know that [they're] terribly open to other people.

Researchers observed that public comments and questions were “closed down” at times if they were deemed not relevant to agenda items, if they were too subjective, or if time was running short.

During earlier observations of meetings a time-slot was included at the end of each agenda item for public members to give comments and ask questions, allowing them to comment directly on the item under current discussion. In later observations a 10 min time-slot for public questions and comments was relocated to the very end of meetings. This meant public members had to wait until the end of the meeting to comment, by which time discussion of a given item had already taken place. This was perceived by public members as a way of “saving time”: by moving (thereby reducing) the public time-slot, the board would have longer to discuss agenda items. One public member commented:

The public involvement is confined to 10 min at the end of the meeting […] I think 10 min for people to ask questions is certainly nowhere near long enough.

Eventually the arrangements for public involvement changed again. The board asked for questions to be submitted in writing ahead of meetings; these would receive priority over verbal questions on the day. Sometimes, however, meetings over-ran and there was insufficient time for responses to written questions. On these occasions, the board announced they would respond in writing at a later date. This was seen as unhelpful by one public member because:

[…] it means all the people here [at the board public meeting] don't get to hear what others are concerned about and the board's response.

### Patient and Public Involvement Clinical Reference Group

Researchers observed two dimensions to the work of the PPI CRG: (i) facilitating development of greater PPI across the borough (e.g., by ensuring on-going communication with and support for other CRGs relating to building PPI) and (ii) supporting the CCG in gaining public feedback about healthcare service development plans (e.g., via public consultations and rolling out public surveys).

The PPI CRG terms of reference stated the overall purpose of the group was to “ensure effective PPI and to deliver to the CCG a vision for PPI.” However, the document lacked detail and was unclear in terms of the role of group members, for example expected achievements and who they were representing and informing—questions that were repeatedly raised by PPI CRG lay members during meetings.

#### Control

Membership to the PPI CRG was controlled by CCG staff regarding the attributes required of new lay members, who would occupy a leadership role and what leadership involved.

Lay members of the PPI CRG had undergone a formal recruitment process to become members of the group. This had involved completing and submitting an application form and CV and attending interview with the lay chair and two or three staff members of the group. Lay members on the whole had previous experience and knowledge of NHS services—some of them in a professional capacity—not only from the patient perspective but also through involvement in other voluntary and community groups.

During researcher observations of PPI CRG meetings two lay members consecutively occupied the role of chair, the first leaving the position when research observations were at an early stage. The second chair was a lay member of the PPI CRG and applied for the position through a formal application process. Senior CCG board members had conducted interviews and appointed the lay member as chair. This appointment automatically afforded the lay member a position on the CCG board as representative for PPI.

The interim period between the first chair leaving the role and recruitment of the second chair was managed by staff members of the group who took on a leadership role and chaired meetings. This received mixed reactions from lay members who, on the one hand, recognized and appreciated the commitment and support of staff members. On the other hand, lay members were at times dissatisfied with the way the group's work and meetings were “managed.” One lay member spoke about feeling:

[…] a bit uncomfortable about the power balance between the staff and the punters.

Notwithstanding this, lay members acknowledged the accountability of staff members to the CCG. Part of the staff's role was to provide written documents (e.g., PPI CRG reports, with input from lay members) about the group's work in developing PPI across the borough. Researcher observations noted perceptions of staff members feeling under pressure when deadlines for producing documents to the CCG board were approaching.

### Valuing Patient and Public Involvement

#### Accountability and Feedback

Lay members expressed a need for stronger lines of accountability in both directions between the PPI CRG and the CCG. Some lay members felt that PPI should be audited to monitor and facilitate progress. This would go some way to strengthening lines of accountability, in turn enabling greater recognition, and value to be placed on PPI:

I do bang on about outcome measurement because I always want to have some demonstration be it qualitative or quantitative that there is a difference being made to actions and outcomes as a result of the conversations that we're having.

However, during observations of PPI CRG meetings, the group did not receive any feedback from the CCG about the work the group had been involved in. One interviewee reported that the only feedback the PPI CRG had received related to the group's written report on developing PPI. The board was interested to know how PPI in the CCG was developing compared with other CCGs and shared the view that PPI in the area was “far ahead in all respects in patient and public involvement.” Lay members expressed a need for meaningful feedback from the CCG:

We don't get much sense when we've done these reports, of has it made any difference […] We keep on trying [to make a difference] without taking stock of whether anybody's hearing what we're saying […] but it would've been quite nice to have got something back from those around us about whether or not they've found anything we've ever done of any use to them.

### Patient and Public Input

#### Linking With the Clinical Commissioning Group

Some lay members had stronger relations with the CCG board, in turn greater levels of input, than other lay and public members. The Healthwatch lay representative and another Healthwatch colleague would meet with the board chair and the chief executive approximately every couple of months “in a private forum” to discuss any issues regarding PPI that came to the representative's attention outside of public meetings:

So in a sense that's not good that the public is excluded from that small meeting but it does help to put across the thoughts and ideas that are coming up from the public through the Healthwatch. They all seem to get a chance to get their message across—why commissioning this or not commissioning that.

The PPI CRG lay chair was also able to speak informally with the CCG board chair and chief executive:

[…] all sorts of conversations take place outside the board and not just involving me, other people have contact with staff, executives or someone […] most of it less than formal meeting level.

The dual role of the PPI CRG lay chair, as a voting board member also, meant they were able to “keep the PPI CRG informed of the bigger picture” regarding the CCG's work and to act as a link between the two. They were also able to give comments on agenda items under discussion at CCG board public meetings and ask questions relating to PPI from their own perspective and/or on behalf of other PPI CRG lay members.

The approach and involvement in the CCG of the PPI CRG lay chair was greatly valued by staff and lay members alike. The lay chair's extensive knowledge and experience and the way they were able to support the development of PPI moving forward, was held in high regard.

#### Influence

Public members who attended CCG board public meetings appeared to have considerably less input to the CCG's work than PPI CRG lay members. One of the reasons relates to the way public involvement in meetings was managed. Another possible reason was that their input may have been viewed as less legitimate—there were no formal recruitment processes to participation. Public members were “independent” and came from a range of backgrounds, particularly different to the backgrounds of PPI CRG lay members. Some public members spoke from personal experience of healthcare services as patients or carers, some spoke from particular political standpoints and most had no “professional” experience of NHS structures and processes.

There were differences in how and at what stage public and lay members wanted to be involved in the CCG's work and how and when the CCG wanted them to be involved. This led to some frustration about the type of involvement lay and public members could or should expect to have. At times PPI CRG lay members perceived their input as “low level”:

Some discussions have come to the [PPI] reference group for input at an early stage […] but they tend to be slow-burning, less high profile issues.

[…] the input was looking at the types of questions that were being asked like, “is this questionnaire okay?”

Some lay members had volunteered their involvement in a commissioning subgroup in order to bear some influence at a higher level, but their involvement had come to an end when, after one meeting, the subgroup stopped meeting.

Lay members wanted to influence commissioning decisions and for the public more broadly to do the same. However, it was emphasized by two staff members that after “essential” healthcare services costs were factored in by commissioners there was very little finance remaining “to play around with.” It was also pointed out that allocating limited resources was a significant responsibility which involved a great deal of skill and particular experience. Based on these restrictions, irrespective of the CCG's approach to PPI, this suggests public and lay members could not have as much input to commissioning decision-making as they believed was or should have been possible.

#### Decision-Making

Researchers did not observe CCG strategic decision-making taking place; agenda items in meetings observed tended not to require decision-making at a strategic level either with or without PPI. CCG board public meetings facilitated discussion amongst board members and other staff, with some input from public members (e.g., around hospital bed arrangements; patient number increase and capacity to meet healthcare needs; plans for patient self-management of clinical conditions; expenditure issues). CRG PPI meetings tended to discuss and respond to CCG requests to build greater PPI more broadly across the borough.

Lay and public members expressed doubts about being heard in a way that made a difference to CCG decision-making at a strategic level including around procurement, commissioning, and future priorities and developments of healthcare services:

I want to know really where people are having an influence and making changes for the better […] but I can't actually get a grasp of where any of us have actually managed to influence spending decisions.

I don't feel we're very influential—I really don't.

For some, the CCG would take notice of PPI only if views matched those of the CCG's:

If it works in the favor of the CCG they'll love it, otherwise they don't want to know.

Public members who attended CCG board public meetings felt that this was not where decision-making in reality took place. It was pointed out that public members were invited to ask questions “which is great” but that:

[…] decisions are obviously made somewhere behind the scenes and they come to the board for ratification.

CCG board public meetings were viewed as a means to public members hearing about changes to services the CCG was planning, but that decisions around those changes had likely already been taken.

## Discussion

Researchers' observations of meetings, interviews conducted, and informal interactions provide insight into the approach, views, and attitudes toward and experiences of PPI in the CCG. Data reveal there are different layers to PPI which reflect different lay and public members' capacity to influence the CCG's work. These layers form part of a hierarchy in which professionals occupy the most powerful positions.

Researchers identified two co-existing dimensions to power. One relates to CCG power over PPI and the other relates to having power to influence the CCG's work. If the CCG has power over PPI, it is reasonable to assume this will affect the nature and extent of PPI input. However, our findings reveal that the different positions of individuals afford them different levels and types of input. Our discussion below considers power imbalances between different individuals and the positions they occupy with regards both dimensions of power: the power over PPI and the power to influence CCG decision-making.

### Power Over Patient and Public Involvement

Issues emerging from our study relating to imbalances of power support findings of previous research regarding the control and restrictions of statutory bodies over public involvement (Baker, 2007; Stern and Green, 2008; Peckham et al., 2014).

Much of the CCG's PPI could be considered low level. Consultation-type PPI is widely recognized as a low level form of involvement (Hickey and Kippling, 1998; Renedo and Marston, 2011). Callaghan and Wistow (2006) found that health boards were viewed as controlling which of its public consultation findings to respond to and concluded that consultation is used to confirm the dominant (professionals') views and not necessarily the public's views. This corresponds with the views of some public and lay members in our study who believed that the CCG would act on PPI only if it corresponded with its own plans.

Direct PPI in CCG decision-making was not evident and neither was there feedback to the PPI CRG from the CCG or outcome measurement of actions resulting from PPI CRG discussions. CCG board public meetings might have intended public participation but in reality there was relatively little. These factors made public and lay members feel underutilized and undervalued highlighting a further aspect of power inequalities. Tritter's (2009) framework for conceptualizing PPI helps us understand the nature of PPI in our study and the power dynamics between professionals and public/lay members. It comprises three dimensions: direct/indirect (the degree of direct decision-making around healthcare service development); individual/collective (the extent that patients and public act as sole agents or as part of a group); proactive/reactive (how much PPI is responding to a pre-existing agenda or is helping to shape it) (2009 p. 277). The PPI in our study can be considered indirect (there was no evidence of direct involvement in decision-making); it is both individual and collective (individual referring to public members at CCG board public meetings and the PPI CRG as the collective). Finally it is both reactive and proactive: public members at CCG board public meetings were reactive because of their capacity to respond only to meeting agenda items and in allocated ways and times. The PPI CRG was reactive in the broad context of the CCG's PPI agenda which determined the nature and level of involvement in the CCG's work. The group was proactive, however, in shaping the PPI agenda to develop PPI more broadly across the borough. Yet even this proactive dimension was conducted under the supervision of the CCG through the support, leadership (at times), and reporting back to the CCG by PPI CRG staff members. Our findings support Tritter's point about the power of professionals to both influence the legitimacy of PPI and limit the type of involvement. Professional support is both an enabler and a restrictor (Tritter, 2009).

### Power to Influence: A Stratified System

The varying degrees of power held by different individuals to influence the CCG's work reflect a system of stratification. Kerckhoff (2001) makes a useful distinction between stratification as a condition and as a process:

Social stratification as a condition refers to the fact that members of a population have characteristics that differentiate them into levels or strata. Social stratification as a process refers to the ways in which members of a population become stratified (Kerckhoff, 2001, p. 3).

In our study, social stratification as a condition relates to the differences in knowledge, qualifications, and experience between professionals and lay members. As a process it relates to how the CCG decides the type and level of PPI afforded to public and lay members.

**Figure 1:**
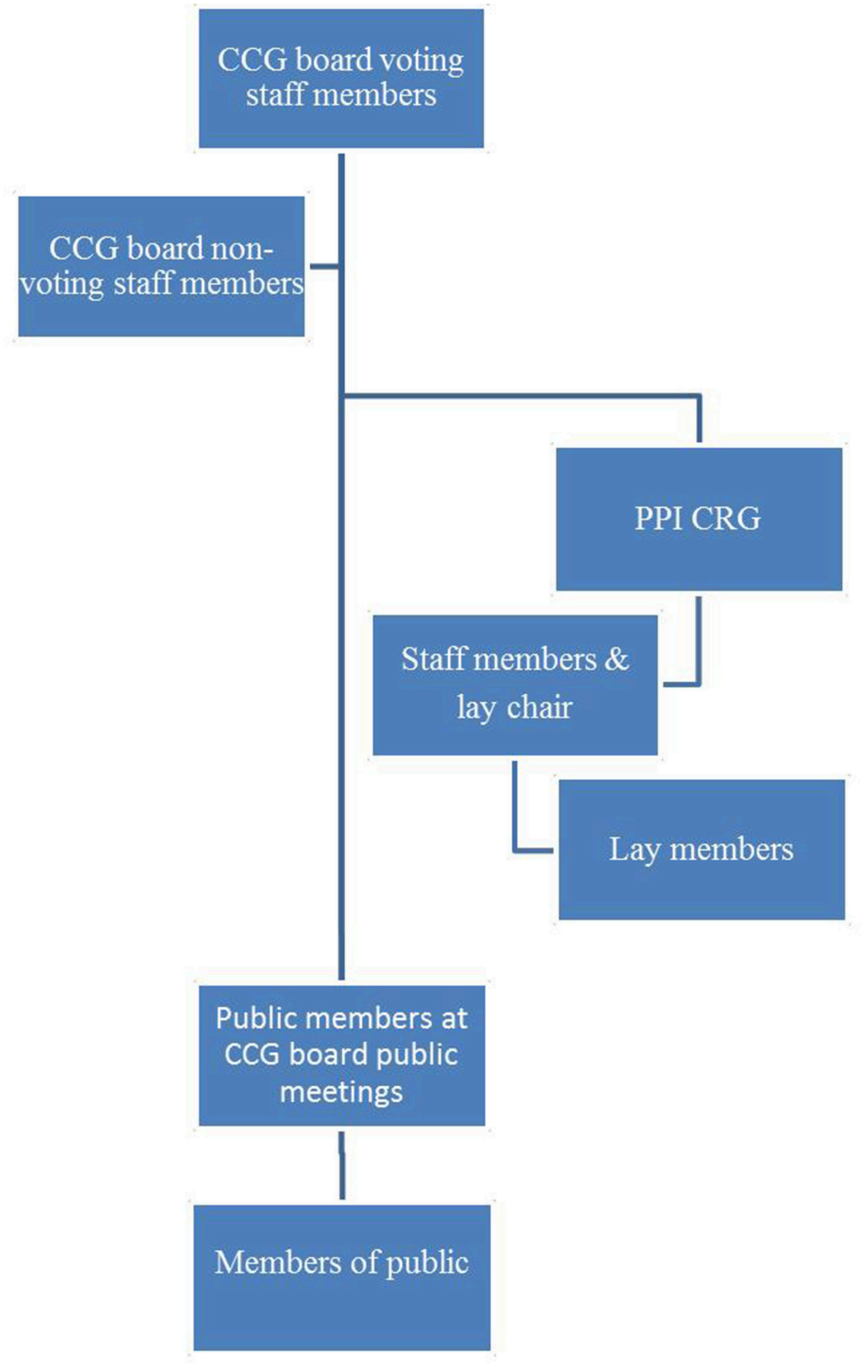
Hierarchy of CCG staff and public/lay member involvement.

The stratification system constitutes a hierarchy of power. Different positions occupied by professionals and public and lay members reflect different levels of power ownership. Power imbalances existed not only between professionals and lay members (although this distinction was the most pronounced), but also amongst different CCG staff and board members and amongst different lay members (Figure 1).

Situated at the top, occupying the most powerful position, were the CCG's 15 voting members. These included two lay members: the PPI CRG chair, and a lay member for governance. Non-voting board members occupied a position below voting members; one of these was the Healthwatch representative. By virtue of sitting on the board, non-voting members nonetheless had the resources to give input and raise questions. Together with other staff members (managers and colleagues) not on the board they were accountable to voting board members when producing service development proposals for approval.

Further down the hierarchy were the CRGs. Within the PPI CRG itself a sub-stratification system or hierarchy existed, starting at the upper end occupied by the lay chair and staff members. The lay chair was a voting CCG board member which automatically afforded a more prominent position, not least by having direct access to CCG board members. They were also involved in separate, smaller meetings with board members outside of CCG board public meetings. Staff members occupied a position somewhere in between the lay chair and other lay members of the group: they were not members of the board but they chaired PPI CRG meetings when there was no lay chair in post. In liaison with the lay chair, they also regulated PPI CRG meetings and agendas.

Other lay members sat on the lower end of the PPI CRG sub-stratification system. They did not have equal access to, or involvement with, CCG board members in comparison to the lay chair and staff members. The privileged position of the lay chair as a CCG board voting member combined with the wider group's involvement in developing broader PPI, its written reports and other tasks requested by the CCG, meant that the PPI CRG as a whole occupied a higher position in the hierarchy than other types of public involvement observed.

In the lower echelons of the hierarchy was involvement from public members who attended CCG board public meetings but whose participation had less potential to influence the CCG's work.

Situated at the bottom of the hierarchy were members of public who did not sit on committees, belong to a formal CRG or attend CCG board public meetings, but took part in CCG public activities, for example consultations. On the one hand, these members of public had least capacity to influence the CCG's work. On the other hand it is possible that public consultations bore some influence on discussions around service development. However, researchers did not observe decision-making taking place. Neither were the CCG's public consultations findings accessed. Without these data we can neither confirm nor refute this point.

Overall, what we see is an example of a hierarchy of power in which, predictably, professionals occupy positions at the top and public members at the bottom. However, we suggest this system has complex dimensions. The dual or overlapping roles of lay members and professionals place them simultaneously on different hierarchical levels. For example the lay chair occupies a position near the top as a voting board member, whilst also being a lay member of the PPI CRG which occupies a lower position. Similarly, staff members of the PPI CRG occupy the same hierarchical position as lay members. Yet as CCG employees with access to senior professionals, and who to a large extent lead and make decisions about the PPI CRG, they also occupy a higher status than their lay member colleagues of the same group.

The hierarchy also demonstrates how positions occupied reflect both the individual and collective dimensions of Tritter's model at the same time. Also in relation to Tritter's model, PPI in CCG board public meetings is at an individual level and at the lower end of the hierarchy while PPI CRG lay members function at a collective level and are positioned higher up. The lay chair of the (collective) PPI CRG, however, is also involved at an individual level but occupies one of the highest positions in the hierarchy. This indicates that having greater or lesser influence is not determined by whether involvement is individual or collective. Further, the only individuals with direct involvement in decision-making are CCG board staff members. Other individuals' involvement may best be described as indirect.

### Structure and Power

The structure and organization of PPI in the CCG was an important factor in governing the flow of power. In order to help inform our interpretations of this finding it is useful to draw on theoretical perspectives.

Power is a core theoretical construct in the field of sociology. It has been a key area of interest in sociological analysis and the impact of power relations on individuals, groups, and organizations over many years.

Paradigms have naturally evolved and shifted in sociology from the 1960s to current day. During the 1960s structural functionalism was a dominant theoretical perspective which attracted critics (e.g., symbolic interactionist Herbert Blumer) for its emphasis on maintaining social orderliness within social and cultural structures, sustaining pre-existing social inequalities and the power of established elite groups. In structural functionalist theory, social stratification is a functional necessity. Stratification as a structure refers to a system of positions (as opposed to individuals in the stratification system) which contain different levels of status. Symbolic interactionism theory criticized the model of structural functionalism for its disregard of individual creativity and micro-level social processes (Cockerham, 2013).

The realm of symbolic interactionism helps to identify the important interaction between structure, culture and action, and provides significant understanding into the relational nature of power, not only in terms of macro structures but also with regard to micro structures and the individual roles that people play within more established organized structures. It is widely recognized that social structures can fashion and influence social interactions and that social interactions can influence, reproduce, and sometimes alter social structures (e.g., Giddens, 1984; Turner, 1992). Established structures, such as macro structures like health organizations, have the potential to promote actions and behaviors among individuals which in turn promotes them to form and continue relationships or affiliations with the dominant structure.

The post-structuralism movement acknowledges the importance of structures. Theorist Michel Foucault, widely associated with post-structuralism, focuses on power and specifically the link between knowledge and power within such structures. Foucault (1980) describes hierarchical bodies and powerful social and cultural structures as having far-reaching, controlling impacts, stretching out and working through every situation in which individuals find themselves (Clark, 2010). Foucault's work reflects how dominant groups, professions and organizations can control agendas to promote or protect their dominance.

The structure and organization of PPI in the CCG reflects a top-down model in which the CCG (the macro structure) is the dominant system and PPI represents social processes at the micro level. The CCG is formed of a board of members which largely comprises medical professionals. Other professionals such as directors and managers of various services also make up board membership. In keeping with Foucault's perspective of power residing in medical structures, institutions and discourses, our findings indicate that the professional status of CCG board members, or the CCG as a structure, places professionals at the top of the hierarchy affording them power over all aspects of the CCG's work. The status of professionals gives them power as professionals in the realm of healthcare services and provisions. Lay and public members, in contrast, are not professionals in this realm; they are recipients of healthcare services and provisions. Attempts are made by the CCG to work collaboratively with lay and public members, but ultimately the professionals represent the dominant structure.

Features of the dominant structure are apparent such as centralized decision-making which appeared to be taking place behind doors that were closed to the public. Although meetings observed were not overly bureaucratic, the CCG board also controlled arrangements around PPI, exemplified for example by the CCG board in public meetings determining when the public could speak, what they could speak about and for how long. By controlling PPI, the status quo is maintained. However, this is not to say the CCG maintained such a level of control in order to promote or protect its dominance. Such an organization with responsibility for making impactful and complex decisions around healthcare service provision would need a level of social orderliness in order to achieve outcomes necessary to provide a service that meets bureaucratic and practical demands.

In theory, the structural model of PPI meetings potentially offered a more collaborative approach to the work of the CCG. In theory, our study was potentially observing a decentralized model through which the public could have strategic input into local healthcare service development. Yet in reality a centralized system governed, facilitating a hierarchy in which the right of the CCG to determine strategy continued largely unchallenged. Lay members showed on-going commitment and support toward (or at the very least involvement in) the work of the CCG which might somewhat reflect a propensity to reinforce power structures through their already established affiliation with the organization.

### Holders of Knowledge

Findings indicate a relationship between knowledge and power when it comes to who can become involved in the work of the CCG, at what level and the extent of influence individuals can have. Foucault's critique of dominant power and knowledge and the disparities between those who possess these resources (professionals) and those who do not (non-professionals) is particularly relevant.

The many different sources and types of knowledge around healthcare [e.g., clinicians', patients/carers', research or evidence-based (Rycroft-Malone et al., 2004)] do not receive equal status and give rise to battles over power and control between competing forms (Shortall, 2012) with professionals questioning the legitimacy of public knowledge (Callaghan and Wistow, 2006). Gibson et al. (2012) emphasize that lay people even on committees are not seen as equal as they do not have the same access to resources as professionals. Whilst our study showed no evidence of professionals questioning the legitimacy of lay members, the more marginal role of lay members in the work of the CCG suggests an unequal balance of potential to influence and ultimately of power between professionals and lay members.

The issue of power imbalances between the different groups of individuals involved in the CCG can be explained by ownership of particular resources, relating to Gibson et al.'s point. CCG board members have professional status. The resources they possess include professional qualifications, knowledge, and expertise. It is ownership of these resources that determines professionals' positions, and it is through these relative positions that they establish levels of power greater than that of other CCG staff and lay and public members. Other CCG staff members possess qualifications, knowledge and expertise but in different areas and at different levels to board member professionals. Lay and public members may also have qualifications, knowledge and expertise but again in areas different to those of the CCG board and to other staff members and, importantly, with less relevance to the CCG's work. When it comes to power imbalances—or more precisely differences in levels of influence—between lay and public members, the former are perhaps viewed as having greater legitimacy because of the particular resources (knowledge, skills, experience, and in some lay members, professionalism) they bring to the CCG. Procedures for recruitment to the PPI CRG suggest that lay members were “cherry-picked” on the basis of these resources. Power imbalances ultimately relate not only to the professional status of individuals and the level and type of resources they possess, but also to perceptions of the legitimacy and relevance of those resources to the CCG's work, and to the access of other individuals to the more powerful professionals.

The position of individuals in the CCG hierarchy is largely governed by professionals' perceptions of lay members' knowledge and based on what type of knowledge is valued. This affords some lay members a higher status (as in the case of the lay chair) but not equal to that of the professionals due to the relativity of the positions they occupy. Where lay people are metaphorically placed is determined by those in positions of power (the professionals) because it is they who control PPI. As Callaghan and Wistow (2006) emphasize, barriers to power-sharing include the beliefs held by professionals about what participation can contribute. A higher position in the CCG hierarchy might enable different PPI and even a greater level of involvement, but does not necessarily translate into a system of equality between the professionals and non-professionals when it comes to influencing the work of the CCG.

## Limitations and Strengths

A main aim of our study was to explore PPI in CCG decision-making. However, we were unable to yield data relating specifically to this due to CCG arrangements around decision-making and around PPI which appeared not to combine the two. It is important at this point to note, though, that our study focused on two particular areas in which there was PPI and not all CCG PPI activities; exploration of other PPI activities may have generated different findings. One way of potentially establishing PPI in strategic decision-making would have been through accessing data from CCG public consultations. These data would then have needed to be compared with decisions the CCG had subsequently taken on the same issues that public consultations addressed, thus becoming more of an evaluation of PPI than an exploration.

A further issue relates to the scale of the study. As a single case study, generalizations about the commissioning arrangements of other CCGs on a national and international level are limited.

The majority of data relating to power derived from researcher observations of meetings and from interviews with public and lay members. Relatively little came from staff interviews. However, the data yielded provide insight into how two types of PPI activity operated in a CCG in England and the views and experiences of many individuals involved.

Contemplating PPI in the context of a stratification system helps us understand the relative position and value accorded to PPI, the different layers to PPI and the levels and types of PPI within those layers. It is appropriate to acknowledge, however, that the hierarchy we present here comprises only those groups and individuals that we observed and conducted interviews with. There would indisputably be other groups and individuals within the CCG (staff and lay members) who would also occupy positions in the hierarchy; it is not by any means exhaustive.

## Concluding Comments

We suggested in our introduction that modern day changes to the way healthcare services are sought, delivered and have facilitated the growth of PPI might have narrowed the power gap between professionals and lay and public members. Consistent with theory and previous research, however, our findings support the premise that professionals hold the most power and therefore continue to dominate; PPI is unable to permeate healthcare commissioning and procurement at an equal level. PPI might have become more integrated into healthcare service development but it still has less status than that of professionals, hence the potency of PPI remains questionable.

The main facilitator of PPI in our study relates to the support provided by the CCG in terms of the functioning of various PPI activities and groups, and the provision of staff and admin support. The barriers to PPI are less tangible but are linked to the legitimacy of public knowledge, an issue that has been widely referred to as restricting effective involvement (e.g., Martin, 2008b; Barnes and Schattan Coelho, 2009; Renedo et al., 2015). It is knowledge (the legitimacy of which is determined by the CCG) and recruitment (which is controlled by the CCG), which are interlinked, that appear to determine where individuals in our study sit in the hierarchy.

We suggest that another related factor of substantial importance when it comes to PPI and power to influence is communication. Effective and on-going communication between public and lay members and professionals could generate greater potential to make public and lay members feel more valued. It could also, importantly, facilitate clarity on all individuals' expectations of the type and level of PPI. An overall more collaborative approach to developing a PPI role which meets the expectations of patients, public, and professionals might go some way toward reducing the power gap between them.

Previous research highlights the need for formal evaluation or monitoring of PPI whilst also underlining associated complexities (e.g., Staniszewska et al., 2011; Brett et al., 2012; Petsoulas et al., 2014). Evaluation would facilitate greater understanding of the strengths of PPI and areas where it could be further developed, in turn enabling greater potential for PPI to have a direct influence on strategic decision-making. In this sense, evaluation of PPI could be an important contribution to narrowing the power disparity between professionals and public and lay members.

## Ethics Statement

The study was granted ethical approval on 14/02/14 by the East of Scotland Research Ethics Service. Permission for conducting observations was obtained from the clinical commissioning group, the patient and public lead, and from lay members of meetings. Informed consent was gained in writing from individuals who participated in interviews and the focus group.

## Author Contributions

All authors contributed to the design of the study and the data collection and analysis activities. AO produced an initial draft of the manuscript, in on-going discussion with AB and MC. All authors contributed significantly to the development and revision of the manuscript and have approved the final version.

### Conflict of Interest Statement

The authors declare that the research was conducted in the absence of any commercial or financial relationships that could be construed as a potential conflict of interest.

## Footnotes

^1^Available online at: https://www.england.nhs.uk/publication/patient-and-public-participation-policy/ (accessed September 10, 2018).

## References

[B1] Baker A. (2007). Patient involvement in a professional body: reflections and commentary. J. Health Organ. Manag. 21, 460–469. 10.1108/14777260710778970 17933376

[B2] Barnes M. Schattan Coelho V. (2009). Social participation in health in Brazil and England: inclusion, representation and authority. Health Expect. 12, 226–236. 10.1111/j.1369-7625.2009.00563.x 19754687 PMC5060493

[B3] Boivin A. Currie K. Fervers B. Gracia J. James M. Marshall C.. (2010). Patient and public involvement in clinical guidelines: international experiences and future perspectives. Qual. Saf. Health Care 19:e22. 10.1136/qshc.2009.034835 20427302

[B4] Brett J. Staniszewska S. Mockford C. Herron-Marx S. Hughes J. Tysall C.. (2012). Mapping the impact of patient and public involvement on health and social care research: a systematic review. Health Expect. 17, 637–650. 10.1111/j.1369-7625.2012.00795.x 22809132 PMC5060910

[B5] Callaghan G. D. Wistow G. (2006). Publics, patients, citizens, consumers? Power and decision making in primary health care. Public Adm. 84, 583–601. 10.1111/j.1467-9299.2006.00603.x

[B6] Cheng J. T. Jessica T. L. Foulsham T. Kingstone A. Henrich J. (2013). Two ways to the top: evidence that dominance and prestige are distinct yet viable avenues to social rank and influence. J. Pers. Soc. Psychol. 104, 103–125. 10.1037/a0030398 23163747

[B7] Clark F. A. (2010). Power and confidence in professions: lessons for occupational therapy. Can. J. Occup. Ther. 77, 264–269. 10.2182/cjot.2010.01.77.5.2 21268508

[B8] Cockerham W. C. (2013). Sociological theory in medical sociology in the early twenty-first century. Soc. Theory Health 11, 241–255. 10.1057/sth.2013.12

[B9] Dahl R. A. (1961). In Rutar, T. (2017). Clarifying power, domination, and exploitation: between “classical” and “foucauldian” Concepts of Power. Revija Za Sociologiju. 2, 151–175.

[B10] Daykin N. Evans D. Petsoulas C. Sayers A. (2007). Evaluating the impact of patient and public involvement initiatives on UK health services: a systematic review. Evid. Policy 3, 47–65. 10.1332/174426407779702201

[B11] Department of Health (2012) Summary Report: Issues Relating to Local Healthwatch Regulations. Available online at: https://assets.publishing.service.gov.uk/government/uploads/system/uploads/attachment_data/file/216865/Summary-Report-Issues-relating-to-local-Healthwatch-regulations.pdf (accessed January 6, 2019).,

[B12] Foucault M. (1972). The Archaeology of Knowledge. London: Tavistock.

[B13] Foucault M. (1980). Power/knowledge: selected interviews and other writings, 1972-1977, ed Gordon C. (New York, NY: Pantheon), 122.

[B14] Foucault M. (2003). The Birth of the Clinic. London: Routledge (original work published 1963).

[B15] Gauvin F.-P. Abelson J. Giacomini M. Eyles J. Lavis J. N. (2010). “It all depends”: conceptualizing public involvement in the context of health technology assessment agencies. Soc. Sci. Med. 70, 1518–1526. 10.1016/j.socscimed.2010.01.036 20207061

[B16] Gibson A. Britten N. Lynch J. (2012). Theoretical directions for an emancipatory concept of patient and public involvement. Health 16, 31–547. 10.1177/1363459312438563 22535648

[B17] Giddens A. (1984). The Constitution of Society: Outline of the Theory of Structuration. Cambridge: Polity Press.

[B18] Guzzini S. (2005): The concept of power: a constructivist analysis. Millennium 33, 495–521. 10.1177/03058298050330031301t.

[B19] Hickey G. Kippling C. (1998). Exploring the concept of user involvement in mental health through a participation continuum. J. Clin. Nurs. 7, 83–88. 10.1046/j.1365-2702.1998.00122.x 9510712

[B20] Institute of Medicine (US) Committee on Quality of Health Care in America (2001). Crossing the Quality Chasm: A New Health System for the 21st Century. Washington, DC: National Academies Press (US). 25057539

[B21] Kerckhoff A. C. (2001). Education and social stratification processes in comparative perspective. Sociol. Educ. 74, 3–18. 10.2307/2673250

[B22] Martin G. (2008a). ‘Ordinary people only’: knowledge, representativeness, and the publics of public participation in healthcare. Sociol. Health Illn. 30, 35–54. 10.1111/j.1467-9566.2007.01027.x 18254832

[B23] Martin G. (2008b). Representativeness, legitimacy and power in public involvement in health-service management. Soc. Sci. Med. 67, 1757–1765. 10.1016/j.socscimed.2008.09.024 18922611

[B24] Mockford C. Staniszewska S. Griffiths F. Herron-Marx S. (2011). The impact of patient and public involvement on UK NHS health care: a systematic review. Int. J. Qual. Health Care 24, 28–38. 10.1093/intqhc/mzr066 22109631

[B25] Naylor C. Curry N. Holder H. Ross S. Marshall L. Tait E. (2013). Clinical Commissioning Groups. Supporting Improvement in General Practice? The King's Fund and Nuffield Trust. Available online at: https://www.kingsfund.org.uk/publications (accessed August 9, 2018).

[B26] Ocloo J. Matthews R. (2016). From tokenism to empowerment: progressing patient and public involvement in healthcare improvement. BMJ Qual. Saf. 25, 626–632. 10.1136/bmjqs-2015-004839 26993640 PMC4975844

[B27] Oliver S. Rees R. W. Clarke-Jones L. Milne R. Oakley A. R. Gabbay J.. (2007). A multidimensional conceptual framework for analysing public involvement in health services research. Health Expect. 11, 72–84. 10.1111/j.1369-7625.2007.00476.x 18275404 PMC5060424

[B28] Peckham S. Wilson P. Williams L. Smiddy J. Kendall S. Brooks F.. (2014). Commissioning for long-term conditions: hearing the voice of and engaging users – a qualitative multiple case study. Health Serv. Deliv. Res. 2:44. 10.3310/hsdr02440 25642540

[B29] Petsoulas C. Peckham S. Smiddy J. Wilson P. (2014). Primary care-led commissioning and public involvement in the English National Health Service. Lessons from the past. Prim. Health Care Res. Dev. 16, 289–303. 10.1017/S1463423614000486 25482225

[B30] Pope C. Ziebland S. Mays N. (2000). Qualitative research in health care: analysing qualitative data. BMJ. 320, 114–116. 10625273 10.1136/bmj.320.7227.114 PMC1117368

[B31] Potrata B. (2005). “She did, he said”: the use of ethnography in CAM research. Complement. Ther. Med. 13, 131–138. 10.1016/j.ctim.2005.03.003 16036171

[B32] Raphael D. Bryant T. (2015). Power, intersectionality and the life-course: identifying the political and economic structures of welfare states that support or threaten health. Soc. Theory Health. 13, 245–266. 10.1057/sth.2015.18

[B33] Renedo A. Marston C. (2011). Healthcare professionals' representations of ‘patient and public involvement’ and creation of ‘public participant’ identities: implications for the development of inclusive and bottom-up community participation initiatives. J. Community Appl. Soc. Psychol. 21, 268–280. 10.1002/casp.1092

[B34] Renedo A. Marston C. A. Spyridonidis D. Barlow J. (2015). Patient and public involvement in healthcare quality improvement: how organizations can help patients and professionals to collaborate. Public Manag. Rev. 17, 17–34. 10.1080/14719037.2014.881535

[B35] Rise B. M. Solbjør M. Lara M. C. Westerlund H. Grimstad H. Steinsbekk A. (2011). Same description, different values. How service users and providers define patient and public involvement in health care. Health Expect. 16, 266–276. 10.1111/j.1369-7625.2011.00713.x 21838833 PMC5060665

[B36] Rutar T. (2017). Clarifying power, domination, and exploitation: between “classical” and “foucauldian” concepts of power. Rev. Sociol. 2, 151–175. 10.5613/rzs.47.2.2

[B37] Rycroft-Malone J. Seers K. Titchen A. Harvey G. Kitson A. McCormack B. (2004). What counts as evidence in evidence-based practice? J. Adv. Nurs. 47, 81–90. 10.1111/j.1365-2648.2004.03068.x 15186471

[B38] Shortall S. (2012). The role of subjectivity and knowledge power struggles in the formation of public policy. Sociology 47, 1088–1103. 10.1177/0038038512454950

[B39] Staniszewska S. Adebajo A. Barber R. Beresford P. Brady L. Brett J.. (2011). Developing the evidence base of patient and public involvement in health and social care research: the case for measuring impact. Int. J. Consum. Stud. 35, 628–632. 10.1111/j.1470-6431.2011.01020.x

[B40] Stern R. Green J. (2008). A seat at the table? A study of community participation in two Healthy Cities Projects. Crit. Public Health 18, 391–403. 10.1080/09581590801959337

[B41] Tritter J. (2009). Revolution or evolution: the challenges of conceptualizing patient and public involvement in a consumerist world. Health Expect. 12, 275–287. 10.1111/j.1369-7625.2009.00564.x 19754691 PMC5060496

[B42] Turner B. S. (1992). Regulating Bodies: Essays in Medical Sociology. London: Routledge.

[B43] Weber M. (1978 [1922]). In Rutar, T. (2017). Clarifying power, domination, and exploitation: between “classical” and “foucauldian” concepts of power. Rev. Sociol. 2, 151–175. 10.5613/rzs.47.2.2

